# Development and validation of the Hocus Focus Magic Performance Evaluation Scale for health professions personnel in the United States

**DOI:** 10.3352/jeehp.2019.16.8

**Published:** 2019-04-10

**Authors:** Kevin Spencer, Hon Keung Yuen, Max Darwin, Gavin Jenkins, Kimberly Kirklin

**Affiliations:** 1 Department of Education, Carlow University, Pittsburgh, PA, USA; 2 Department of Occupational Therapy, School of Health Professions, University of Alabama at Birmingham, Birmingham, AL, USA; 3 University of Alabama at Birmingham, Birmingham, AL, United States; 4 Department of Occupational Therapy, School of Health Professions, University of Alabama at Birmingham, Birmingham, AL, USA; 5 UAB’s Institute for Arts in Medicine, Alys Stephens Center, University of Alabama at Birmingham, Birmingham, AL, USA; Hallym University, Korea

**Keywords:** Reproducibility of results, Health occupations, Complementary therapies, United States

## Abstract

**Purpose:**

This study was conducted to describe the development and validation of the Hocus Focus Magic Performance Evaluation Scale (HFMPES), which is used to evaluate the competency of health professions personnel in delivering magic tricks as a therapeutic modality.

**Methods:**

A 2-phase validation process was used. Phase I (content validation) involved 16 magician judges who independently rated the relevance of each of the 5 items in the HFMPES and established the veracity of its content. Phase II evaluated the psychometric properties of the HFMPES. This process involved 2 magicians using the HFMPES to independently evaluate 73 occupational therapy graduate students demonstrating 3 magic tricks.

**Results:**

The HFMPES achieved an excellent scale-content validity index of 0.99. Exploratory factor analysis of the HFMPES scores revealed 1 distinct factor with alpha coefficients ≥0.8 across the 3 magic tricks. The construct validity of the HFMPES scores was further supported by evidence from a known-groups analysis, in which the Mann–Whitney U-test showed significant difference in HFMPES scores between participants with different levels of experience in delivering the 3 magic tricks. The inter-rater reliability coefficients were ≥0.75 across the 3 magic tricks, indicating that the competency of health professions personnel in delivering the 3 magic tricks could be evaluated precisely.

**Conclusion:**

Preliminary evidence supported the content and construct validity of the HFMPES, which was found to have good internal consistency and inter-rater reliability in evaluating health professions personnel’s competency in delivering magic tricks.

## Introduction

Magic has been used clinically as a therapeutic modality to motivate patients to engage in activities to boost motor skills [1,2], to build rapport [3], to gain cooperation [4], and to reduce psychological discomfort, stress, and anxiety [5]. Overall, patients benefit both physically and psychologically from actively and/or passively participating in the art of magic. This is done by actively learning and performing magic tricks for others, or passively by merely watching a live magic performance performed by health care practitioners or students [6,7].

However, performing a magic trick is not simply about knowing the secret or executing the moves. Performers are required to utilize and integrate a variety of skills that result in a masterful performance to create the desired illusion [8,9]. It is also important for the performer to develop his/her performance skills, enabling him/her to enhance the presentation and capture the attention of the audience [8,9]. For magic tricks to be a therapeutically effective modality that is engaging and motivating to the patient, it is essential that the performer deliver them competently.

Methods to evaluate practitioners’ competency have been developed for other creative or expressive art therapy approaches; however, no assessment tool currently exists to evaluate the competency of a health care practitioner or student in delivering magic tricks. The purpose of this study was to develop and validate an evaluation instrument of magic trick performance skills for health care students, referred to as the Hocus Focus Magic Performance Evaluation Scale (HFMPES). The study was conducted in 2 phases; phase I was content validation, and phase II was an evaluation of the psychometric properties of the HFMPES.

## Methods

### Ethics statement

The Institutional Review Board of the University of Alabama at Birmingham approved the study protocol (IRB approval no., 300000286).

### Study design

A cross-sectional survey research design was used for both phases.

### Phase I: Content validation

This phase was conducted to assess the relevance of each item in the HFMPES. The HFMPES was developed by the first author, who is a professional magician and faculty member of the special education program at a university. The HFMPES items were developed based on the first author’s practice and teaching experience of magic (>20 years), as well as recommended principles of magic delivery in the literature [8,9]. The HFMPES consisted of 5 items, including the essential characteristics needed to perform magic tricks effectively and engagingly (Table 1). Each item was rated on a 5-point rating scale (Table 2).

**Table 1.** Item evaluation of the Hocus Focus Magic Performance Evaluation Scale by professional magicians (n=16)

Items	Task	No. of members giving a rating of 4 (%)	Content validity index
1	The coach recalls the steps sequentially without delay.	13 (81)	1.00
	If the steps are not performed in the proper sequence, the trick will not work.		
2	The coach performs each step correctly.	14 (88)	1.00
	Not only is it important for each step to be performed in the proper sequence, it is equally important that each step is performed accurately.		
3	The coach performs each step smoothly.	11 (69)	0.94
	Each step must be performed in sequence and smoothly; however, each step must also be executed in such a way that no one step is more noticeable than the other - everything should appear “natural” or “normal.”		
4	The coach performs the magic trick without exposing the secret.	13 (81)	1.00
	Each step must be done in the right order, well executed and naturally, and without either directly exposing the secret or bringing unnecessary attention to the secret.		
5	The coach presents the trick with personal flair and artistry.	14 (88)	1.00
	Presentation of the trick must be done in a such a way that it is interesting and captures the attention of their client. More than simply a series of moves, it must be presented in a way that taps their curiosity.		

**Table 2.** Definitions of the scoring criteria of the rating scale for the Hocus Focus Magic Performance Evaluation Scale

Rating	Description/definition
0	Never meets expectations
1	Sometimes meets expectations
	Executes the moves sequentially but inconsistently
	Executes the moves correctly but occasionally pauses or delays
2	Meets expectations
	Executes the moves sequentially, correctly, confidently, and skillfully (without pause or delay)
3	Meets but sometimes exceeds expectations
	Executes the moves sequentially, correctly, confidently, and skillfully (without pause or delay)
	Occasionally incorporates a story or patter in the presentation
	Occasionally incorporates appropriate gestures in performance
	Occasionally engages audience with eye contact and social interaction
4	Exceeds expectations
	Executes the moves sequentially, correctly, confidently, and skillfully (without pause or delay)
	Consistently incorporates a story or patter in the presentation when appropriate
	Consistently incorporates appropriate gestures in performance
	Consistently engages audience with eye contact and social interaction

#### Subjects

Twenty magicians were selected by the first author from the database of the 2 largest organizations dedicated to the art of magic—the Society of American Magicians and the International Brotherhood of Magicians—to evaluate the HFMPES items. The selection criteria of these judges were based on some level of acquaintance with the first author, and being professional magicians.

#### Procedures

A letter explaining the purpose of the study with the HFMPES attached was e-mailed to these 20 magicians. The letter requested them to rate the relevance of each of the 5 items in the HFMPES using a 4-point scale: 1=not relevant, 2=unable to assess relevance without major revision, 3=relevant but needs a minor alteration, and 4=very relevant and succinct. Respondents were encouraged to highlight words or portions of the item descriptions that were unclear and to suggest alternate phrasing.

#### Data analysis

Item relevance to the HFMPES was determined by the percentage of agreement among the respondents’ ratings using the content validity index (CVI) [10], defined as the number of judges giving a rating of 3 or 4, divided by the number of judges in the panel. The criterion to retain an item was set at ≥0.8 [10].

#### Outcomes

Sixteen magicians (11 males and 5 females) completed the evaluation, resulting in a 80% response rate. The mean±standard deviation (SD) years of the panel members’ work experience as a magician was 31±13.5 years, and their mean experience of teaching magic was 7±9 years. The scale-CVI of the HFMPES, which is the average of the item-CVI, was 0.99. Four items achieved unanimous agreement, and item 3 had a CVI of 0.94 (Table 1).

The panel members also provided suggestions, albeit minimal, on the wording of the items. For example, a respondent suggested changing the wording ‘finesse’ in item 5, as it was too vague. After considering all comments, the word ‘finesse’ in item 5 was replaced with ‘personal flair and artistry.’ In addition, the definitions of the scoring criteria of each response option on the rating scale were elaborated to facilitate a more accurate rating assignment (Table 2).

### Phase II: Evaluation of psychometric properties

Evaluation of the psychometric properties of the HFMPES involved validation of the factor structure, known-groups analysis, and assessments of the internal consistency and inter-rater reliability.

#### Subjects

Seventy-three occupational therapy (OT) graduate students (40 first-year and 33 second-year) participated in this study. For several years, the first author has been invited to deliver a workshop to OT students on the therapeutic role of magic in rehabilitation in September at the participating university. The second-year students were introduced to magic tricks in 2017, whereas the first-year students were introduced in 2018. Seventeen second-year students also volunteered to participate in a summer magic camp held in June 2018 for 2 consecutive weeks, and coached children with hemiplegia to learn pre-selected tricks.

Of the 33 second-year participants, 15 of them participated in the summer magic camp 5 months prior to this study. The mean±SD age of the 73 participants was 25.3±3.4 years old; 66 were female, 7 were male, 54 were White, 13 were Black, 5 were Asian, and 1 identified herself as other.

#### Procedures

Participants were asked to demonstrate 3 tricks for 2 magicians (the first and third authors) via Zoom videoconferencing. The tricks were previously learned by the students while participating in the workshop conducted by the first author.

Each participant stood behind a table situated approximately 2 m in front of a computer monitor with a mounted webcam. The table contained all the items needed to perform the 3 tricks. Through the webcam, the two magicians could clearly see the participant from head to waist, including every arm and hand movement. The 2 magicians were stationed in different parts of the country, but were able to simultaneously and independently rate each participant’s performance using the HFMPES.

Each participant performed the 3 tricks (challenge knot, linking paper clips, and hopping rubber bands) in the same order in order to avoid potential human errors made by the 2 magicians in recording the rating scores. A detailed description of the 3 tricks is in the Appendix 1. Participants were also encouraged to include their own unique elements in the performance of each trick (e.g., a short story). Each trick presentation took approximately 1 minute to complete.

Videos showing how to perform these 3 tricks were made available to all students a week before the first study session. Participants were reminded to rehearse these 3 tricks beforehand. The study spanned 3 consecutive sessions, with 1 session per day. At the completion of each session, the 2 magicians submitted their scores to the investigative team.

#### Data analysis

Validation of the HFMPES was conducted for each of the 3 tricks separately. The principal axis factoring method was applied to each trick to evaluate the dimensionality of the HFMPES. A known-groups analysis was used to demonstrate that HFMPES scores were able to distinguish between participants who had participated in the summer magic camp and those who had not. In order to control for the recency of exposure to magic tricks, only the second-year students were included when conducting the known-groups analysis. The hypothesis was tested using a 1-sided test with the type I error rate set at 0.05. The Cronbach α was used to estimate the internal consistency reliability. To assess the inter-rater reliability between the 2 magicians’ ratings, intraclass correlation coefficients (ICC [1,2]) were calculated.

Data analysis was based on participants’ scores from magician rater 1 (the first author). Sensitivity analyses were conducted using data from magician 2 to validate the findings. Magician 2 was completely blind to the purpose of the study and participants’ previous experience with magic lessons. The raw data are available in Supplement 1.

## Results

### Factor structure

The number of factors extracted was determined using the eigenvalue >1 rule. An item was retained if its factor loading was ≥0.4. Eigenvalues revealed 1 factor (unidimensionality) of the HFMPES for each of the 3 tricks. The factor accounted for 72.7% of the total variance in the challenge knot trick, 67.1% in the linking paper clips trick, and 79.4% in the hopping rubber bands trick. Similar findings were observed in magician 2’s ratings. Given the unidimensionality of the HFMPES, a composite score was formed by summing the score of all 5 items.

### Construct validity

For the known-groups analysis, the results of the Mann-Whitney U-test indicated that the HFMPES scores for the 3 tricks, as rated by the magicians, of participants who had participated in the magic camp were significantly higher than the scores of those who had not participated, with P-values <0.05 (one-sided) except for the hopping rubber bands trick, rated by magician 2, for which no significant difference was observed between the 2 groups of participants (P=0.19) (Table 3). To confirm that there was no bias in magician 1’s ratings, a comparison of the HFMPES scores between the 2 raters was conducted. The results of the Wilcoxon signed-rank test indicated no significant difference in the HFMPES scores between the 2 magicians for participants who had participated in the magic camp (n=15) across the 3 tricks, with all P-values ≥0.20.

**Table 3.** Comparison of Hocus Focus Magic Performance Evaluation Scale scores between participants who had participated in a summer magic camp and those who had not

Rater	Challenge knot			Linking paper clips			Hopping rubber bands		
	Magic camp group (n=15)	Non-magic camp group (n=18)	P-value (one-sided)	Magic camp group (n=15)	Non-magic camp group (n=18)	P-value (one-sided)	Magic camp group (n=15)	Non-magic camp group (n=18)	P-value (one-sided)
1	16 (13.0–18.0)	10 (6.0–15.0)	<0.001	13 (11.0–16.0)	11 (7.3–13.0)	0.037	11 (8.0–15.0)	8 (4.3–10.3)	0.037
2	13 (12.0–16.0)	10.5 (5.0–13.3)	0.006	13 (12.0–14.0)	9 (6.0–11.5)	0.006	11 (7.0–16.0)	10 (4.8–14.3)	0.19

Values are presented as median (interquartile range). The participants (n=33) in this comparison were all second-year occupational therapy graduate students.

To support the concept that the 3 tricks posed different levels of challenge and demanded different types of motor skills, Table 4 shows a correlation matrix depicting the associations among the HFMPES scores from magician rater 1 for the 3 tricks and compares them to the associations among HFMPES scores of the 3 tricks assigned by rater 1 and rater 2. As indicated, the correlation coefficient was larger between rater 1’s HFMPES scores for the challenge knot and linking paper clips tricks (r=0.51) than between the scores for the challenge knot and hopping rubber bands tricks (r=0.38). The correlation coefficients among rater 2’s HFMPES scores for the 3 tricks were almost identical to those of rater 1 (results not shown). A similar pattern was also observed for the correlation coefficients among the HFMPES scores of the 3 tricks assigned by rater 1 and rater 2.

**Table 4.** Correlation matrix of the association of HFMPES scores from rater 1 for the 3 magic tricks and the associations between the HFMPES scores of the 3 tricks from rater 1 and rater 2

Rater 1	Rater 1			Rater 2		
	Challenge knot	Linking paper clips	Hopping rubber bands	Challenge knot	Linking paper clips	Hopping rubber bands
Challenge knot	-	0.51	0.38	-0.78	0.39	0.25
Linking paper clips		-	0.47		-0.78	0.41
Hopping rubber bands			-			-0.75

The values in parenthesis are inter-rater reliability coefficients. The ICCs of inter-rater reliability between the HFMPES composite scores of the 2 raters for the 3 magic tricks were as follows: challenge knot trick, 0.78 (95% CI, 0.67–0.85); linking paper clips trick, 0.77 (95% CI, 0.65–0.85); and hopping rubber bands trick, 0.75 (95% CI, 0.62–0.83). Using the ICC to compute the associations among the 3 HFMPES scores of the 2 raters for the 3 magic tricks produced the same coefficients in the correlation matrix as using the Pearson product moment correlation. HFMPES, Hocus Focus Magic Performance Evaluation Scale; ICC, intra-class correlation coefficients; CI, confidence interval.

### Internal consistency reliability

The internal consistency reliability coefficients of the HFMPES for the 3 tricks ranged from 0.87 to 0.93 for rater 1’s scores, and from 0.80 to 0.91 for rater 2’s scores. Additionally, the corrected item-to-total correlations between scores of an individual item and the summation score of the remaining items were all above >0.54. Eliminating an item from the HFMPES increased the alpha coefficient slightly in some instances, but by no more than 0.02 points, indicating that all 5 items fit reasonably well with the underlying construct of the HFMPES.

### Inter-rater reliability

The ICCs of inter-rater reliability between the HFMPES composite scores assigned by the 2 raters for the 3 tricks were as follows: challenge knot, 0.78; linking paper clips, 0.77; and hopping rubber bands, 0.75.

## Discussion

The results from the 2-phase validation of the HFMPES support its content validity and psychometric properties as applied to 3 different tricks performed by OT students. With an excellent scale-CVI of 0.99, the content of the HFMPES was considered relevant for evaluating the participants’ skills in delivering magic tricks. Exploratory factor analysis revealed 1 distinct factor, which was conceptually aligned with the underlying construct envisioned by the research team. In addition, the high alpha coefficient value of the HFMPES (≥0.8) as applied to each of the 3 tricks indicated that all 5 items fit well with the underlying construct. The construct validity of the HFMPES was further supported by evidence from the known-group analysis, which indicated that the HFMPES was able to distinguish participants with different levels of experience in delivering the 3 tricks.

The HFMPES was validated for 3 magic tricks, each of which posed a different level of challenge and demanded different types of motor skills. The findings from the correlation matrix on the associations of HFMPES scores assigned by each rater among the 3 tricks and the associations between the HFMPES scores of the 3 tricks from different raters indicated there were more similarities between the challenge knot and linking paper clips tricks and between the linking paper clips and hopping rubber bands tricks, but less similarity between the challenge knot and hopping rubber bands tricks. The high inter-rater reliability coefficients (ICC≥0.75) also indicate that the competency of the participants in delivering these 3 tricks could be evaluated precisely.

The sample sizes for content validation and reliability assessment were deemed to be sufficient. We included 16 judges to conduct the item evaluation, which was more than the recommended number of within 10, and included 73 participants to assess the inter-rater reliability, which exceeds the recommended sample size for the assessment of inter-rater reliability of at least 50 [11].

The HFMPES was designed to assess the important characteristics of one’s ability to effectively perform a magic trick. Each of the 5 items is self-explanatory and does not require a magician to judge a person’s competency in delivering tricks. The HFMPES provides a benchmark of competency for health care providers and students to demonstrate or teach magic tricks to their patients.

The HFMPES was developed to evaluate simple magic tricks. Psychometric properties derived from the performance evaluation of complex tricks may not be as strong as the findings of the present study.

## Supplementary materials

Supplement 1. Data files are available from https://doi.org/10.7910/DVN/2DHW1G.

Supplement 2. Audio recording of the abstract.

## Appendix 1. Description of the three magic tricks

Three magic tricks were selected for the participants to perform for evaluation: challenge knot, linking paper clips, and hopping rubber bands.

For ‘challenge knot,’ the participant ties a knot in the middle of a rope without releasing either end of the rope. To accomplish this, the participant crosses his/her arms before picking up the ends of the rope and then uncrosses them to reveal the knot. This trick requires the participant to follow simple directions, sequence the steps correctly, cross the body’s midline, and use upper body gross motor skills.

For ‘linking paper clips,’ the participant strategically places two paper clips on a one dollar bill and pulls the corners causing the paper clips to jump into the air and link together. This trick requires the participant to follow complex directions, sequence the steps correctly, and use fine motor dexterity, bilaterality, pad to pad prehension, and visual motor integration.

For ‘hopping rubber bands,’ the participant places a rubber band on the first and second fingers and causes it to magically jump to the third and fourth fingers. This trick requires the participant to follow complex directions, sequence the steps correctly, use flexion and extension of the fingers, abduction and adduction of the fingers, pad to pad prehension, wrist rotation, and supination and pronation of the forearm. These three magic tricks offer a different approach to hand-arm bimanual intensive therapy for children with hemiplegia by motivating them to purposefully use their affected hand and arm.
